# Forgotten Goiter Presenting As Acute Respiratory Distress Following a Thyroidectomy: A Case Report

**DOI:** 10.7759/cureus.54933

**Published:** 2024-02-26

**Authors:** Beatriz Marques, Rita Oliveira, Roberto J Ameiro, Manuela Paiva

**Affiliations:** 1 Department of Anaesthesiology, Centro Hospitalar de Vila Nova de Gaia/Espinho, Vila Nova de Gaia, PRT

**Keywords:** head neck surgery, post-op complications, difficult airway management, thyroidectomy complications, forgotten goiter

## Abstract

Airway complications account for a significant amount of post-thyroidectomy complications. Forgotten goiter is a residual thyroid mass left after total thyroidectomy, an event already depicted in the literature. Clinical presentation is diverse, ranging from asymptomatic tracheal deviation to symptoms caused by hormonally active thyroid tissue or airway obstruction due to mass effect. However, it has never been documented as the cause of acute respiratory distress following thyroid surgery. We report the case of a 65-year-old female undergoing left hemithyroidectomy due to long-standing substernal goiter. Anesthesia induction and surgery were uneventful. On extubation, the patient presented with acute respiratory distress requiring prompt airway management. A computed tomography scan revealed residual intrathoracic goiter resulting in significant airway compression. Therefore, although a rare event, a forgotten goiter should be considered by a multidisciplinary team when patients undergoing surgery for substernal goiter develop acute postoperative airway obstruction after common post-thyroidectomy complications have been excluded.

## Introduction

The term goiter refers to the abnormal growth of the thyroid gland. Forgotten goiter is defined as a mediastinal thyroid mass found postoperatively after total thyroidectomy. Substernal goiter is present in 3-20% of surgeries performed due to goiter. Complications such as cervical hematoma, laryngeal edema, tracheomalacia, and recurrent laryngeal nerve palsy are well-documented after surgery. Risk factors include the presence of preoperative compression symptoms, goiter size, and advanced age [1]. The incidence of perioperative complications is usually higher following thyroidectomies performed for substernal goiter [2] and there have been few reports concerning forgotten goiters [3]. We aim to report the first case of acute respiratory obstruction following thyroidectomy due to a forgotten goiter. 

## Case presentation

Patient history

A 65-year-old female, American Society of Anesthesiologists (ASA) physical status III, with a history of morbid obesity (BMI 41), was admitted for left hemithyroidectomy due to long-standing large multinodular substernal goiter. The patient was suggested to undergo surgical treatment, which she had repeatedly refused.

Preoperative assessment

**Figure 1:**
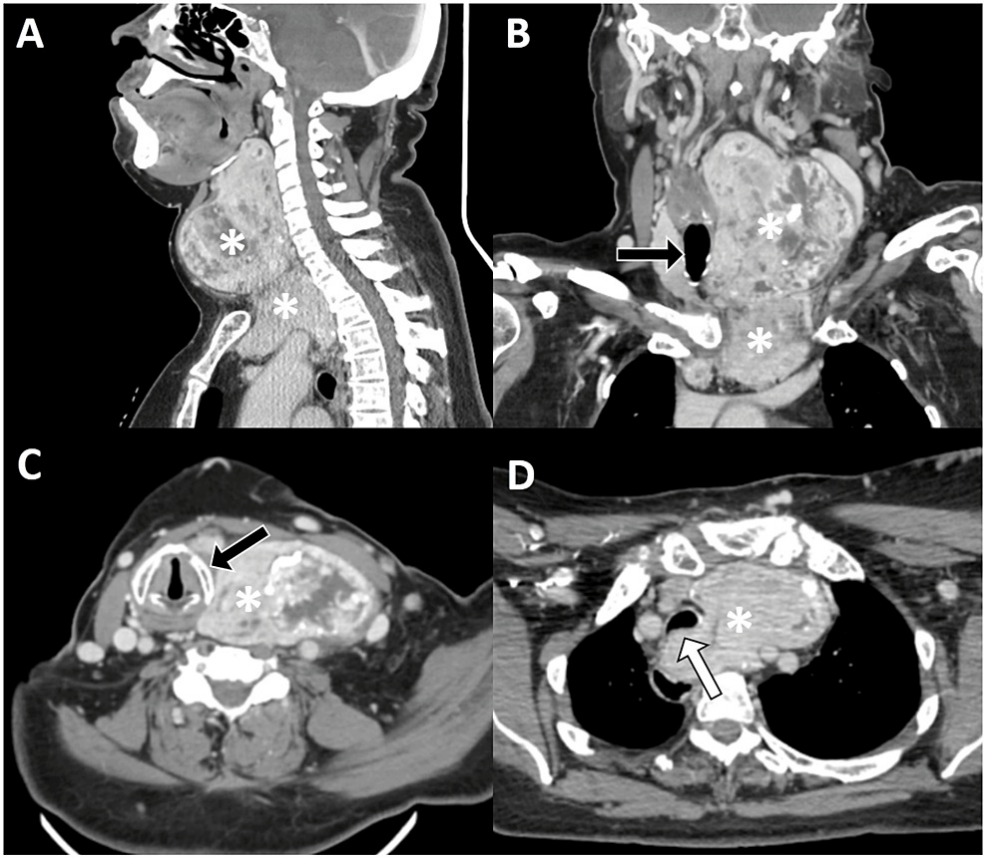
Preoperative CT scan. Saggital (A) and coronal (B) images showed a voluminous left cervical goiter with upper mediastinal extension (asterisks), measuring 10.2 x 6.8 x 15.3 cm (transverse x anteroposterior x longitudinal). Lateral right deviation of the trachea and larynx (black arrow). The axial cervical image (C) demonstrated a left goiter resulting in right deviation of the larynx (black arrow). The axial upper mediastinal image (D) demonstrated compression of the trachea (white arrow).

A preoperative anesthetic evaluation revealed severe cervical dysmorphism. The patient reported worsening compressive symptoms over a two-year long period, such as dyspnea with moderate efforts, dysphagia, and dysphonia. Airway evaluation revealed Mallampati class 3, limited mouth opening, reduced thyromental distance, and a large neck circumference. A preoperative cervical computed tomography (CT) scan (Figure 1) showed hypertrophy of the left thyroid lobe. Thyroid function tests were within normal limits. 

Intraoperative course

On the day of the procedure, the patient was monitored according to ASA standards for invasive blood pressure, Bispectral Index (BIS), and Train of Four (TOF) parameters. In anticipation of potential airway complications, a bronchologist and an otolaryngologist were present.

Awake fiberoptic tracheal intubation (FTI) was performed under light sedation with a target-controlled infusion (TCI) of remifentanil, Minto's model with a plasma target concentration of 2 ng/ml, and topical anesthesia with a 10mg spray of Xylocaine. The patient was collaborative and had stable respiratory and hemodynamic parameters during the procedure. The airway was successfully secured.

Anesthesia induction and maintenance were done using total intravenous anesthesia (TIVA) with propofol and remifentanil TCI (Scnider's model with plasma effect concentration of 5 ug/ml for loss of consciousness followed by maintenance titration by BIS, 2-3 ug/ml plasma effect concentration for propofol; Minto's model with plasma effect concentration of 2 ng/ml for remifentanil), and neuromuscular blockade with rocuronium (initial 60mg bolus followed by 20mg repeated boluses guided by TOF counts >1). 

The surgery lasted approximately two hours and was uneventful. During the emergence of anesthesia, neuromuscular blockade reversal was performed with sugammadex (2 mg/kg) and confirmed with TOF monitoring (repeated three TOF counts ratio >95%).

Immediate postoperative period

Extubation was successfully performed when the patient was fully awake, cooperative, and capable of maintaining a satisfactory tidal volume (7 ml/kg) and respiratory rate (16 cycles per minute). Immediately after extubation, the patient was able to talk without any stridor but, just seconds later, reported worsening dyspnea and presented with diminished response to stimuli. A blood gas analysis revealed significant hypercarbia (PaCO_2_=64 mmHg). The patient's airway was once again secured with awake FTI and the patient was transferred to the Intensive Care Unit (ICU). 

Postoperative complications associated with thyroid surgery were suspected and investigated. There were no signs of tracheomalacia during the fiberoptic assessment. The fiberoptic assessment found edema. 

Postoperative assessment

After arrival in the ICU, on day one, cuff-leak tests were performed. Without an air leak after cuff deflation, these were attributed to edema, which delayed extubation. A chest X-ray revealed no significant findings. On day two, a fiberoptic assessment found that the airway edema receded under corticoid therapy. Further cuff-leak tests were negative.

**Figure 2:**
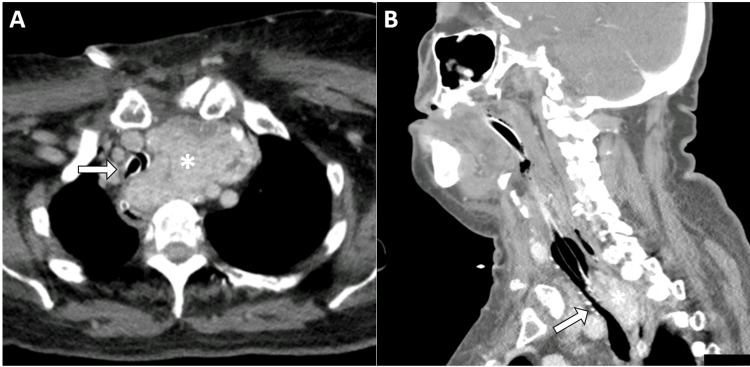
Postoperative CT scan performed after initial surgery. Axial (A) and saggital (B) images showed residual substernal left goiter (asterisks), measuring 8.3 x 5.1 x 5.9 cm (transverse x anteroposterior x longitudinal), causing significant tracheal compression (white arrows).

Due to persistent airway obstruction, further testing was conducted to exclude tracheomalacia or a compressive internal hematoma (although not likely due to hemodynamic and haemoglobin stability). A CT scan performed on day 2 showed a residual intrathoracic goiter fragment (Figure 2). 

**Figure 3:**
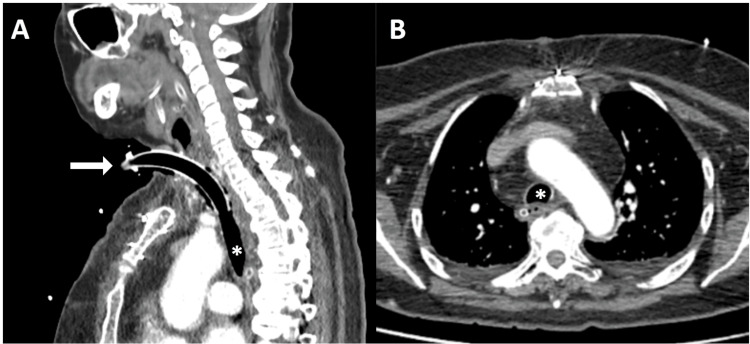
Postoperative CT scan performed after second surgery. Axial (A) and saggital (B) images showed a tracheotomy (arrow) and complete excision of the residual left goiter. The airway was patent with resolution of the tracheal compression (asterisks).

A new surgery was scheduled on day three to remove the remaining thyroid tissue. Complications were not reported and a surgical tracheostomy was performed to prevent further complications. The patient was once again transferred to the ICU. Weaning from mechanical ventilation was initiated on day three. A CT scan performed after the second surgery showed a resolution of the tracheal compression (Figure 3).

By day four, the patient was able to tolerate spontaneous ventilation, and supplemental oxygen was progressively decreased. The patient was disconnected from the ventilator on day four, and by day six, the tracheostomy cannula was removed. The patient made a full recovery. 

## Discussion

Airway complications account for 12.8% of the overall post-thyroidectomy complications. Frequent causes include cervical hematoma, laryngeal edema, tracheomalacia, and recurrent laryngeal nerve palsy [4]. The diagnostic hypothesis first considered in the operating room (OR) was laryngeal edema (documented with fiberoptic assessment) or tracheomalacia. Nerve palsy was discarded because the patient was able to talk after extubation. Cervical hematoma should have been promptly discarded while still in the OR, but the surgical team discarded that complication as there was scant hematic drainage and no signs of an increasing cervical mass. Airway edema was considered the most likely cause. 

During the ICU stay, this assumption was ruled out, as the edema subsided with a course of steroids while the obstruction persisted. Tracheomalacia was also excluded, as there was no evidence in further fiberoptic assessments. 

Hematoma was again considered, as it has been described as a rare but important cause of airway obstruction in the first 24 hours after surgery [5]. An unnoticeable internal hematoma could be possible, compressing the airway and resulting in obstruction. Therefore, a CT scan was performed. 

Forgotten goiter's cases are often detected years following surgical resection, due to symptoms caused by hormonally active thyroid tissue or insidious airway obstruction [2]. To our knowledge, this is the first described case of an acute airway obstruction due to a forgotten goiter in the immediate postoperative period, representing a diagnostic challenge to anesthesiologists and surgeons in the postoperative period, and highlighting the importance of a multidisciplinary approach. 

## Conclusions

This case highlights forgotten goiter as a new possible acute post-thyroidectomy complication resulting in airway obstruction in the immediate postoperative period. It is a well-known possible surgical complication but has never been an acute airway-compromising factor.

In patients undergoing surgery for substernal goiter presenting with acute postoperative airway obstruction in which common post-thyroidectomy complications have been excluded, forgotten goiter should be considered as a possible, yet rare, cause. A careful preoperative assessment of the goiter, considering its substernal extension, should be performed and discussed with the multidisciplinary team. Moreover, an early CT scan following the surgery could help make a prompt diagnosis. 
